# Perceptions and knowledge regarding the COVID-19 pandemic between U.S. and China: a mixed methods study

**DOI:** 10.1186/s12992-022-00864-y

**Published:** 2022-08-08

**Authors:** Yutang Xiong, Xingran Weng, Bethany Snyder, Lin Ma, Menglong Cong, Erin L. Miller, Lauren Jodi Van Scoy, Robert P. Lennon

**Affiliations:** 1 Department of Research Methods and Statistics, Morgridge College of Education, University of Denver, 1999 E Evans Ave, Denver, CO USA; 2 The Frederick S. Pardee Center for International Futures, Josef Korbel School of International Studies, University of Denver, 2201 S. Gaylord St., Denver, CO USA; 3 Department of Public Health Sciences, Penn State College of Medicine, 90 Hope Drive, Hershey, PA USA; 4 Center for Community Health Integration, School of Medicine, Case Western Reserve University, 11000 Cedar Ave, Cleveland, OH USA; 5 Department of Family and Community Medicine, Penn State College of Medicine, 90 Hope Drive, Hershey, PA USA; 6 Department of Medicine, Penn State College of Medicine, 500 University Drive, Hershey, PA USA; 7 Department of Humanities, Penn State College of Medicine, 500 University Drive, Hershey, PA USA

**Keywords:** COVID-19, U.S. and China, Mixed methods, Public messaging, Sociocultural differences

## Abstract

**Background:**

SARS-CoV-2, a new coronavirus first reported by China on December 31st, 2019, has led to a global health crisis that continues to challenge governments and public health organizations. Understanding COVID-19 knowledge, attitudes, and practices (KAP) is key for informing messaging strategies to contain the pandemic. Cross-national studies (e.g.: comparing China to the U.S.) are needed to better understand how trans-cultural differences may drive differences in pandemic response and behaviors. The goal of the study is to compare knowledge and perceptions of COVID-19 between adults in China and the U.S. These data will provide insight into challenges these nations may face in coordinating pandemic response.

**Methods:**

This is a convergent mixed methods study comparing responses from China and the U.S. to a multinational COVID-19 KAP online survey. The survey included five quantitative constructs and five open-ended questions. Chinese respondents (*n* = 56) were matched for gender, age, education, perceived social standing, and time of survey completion with a U.S. cohort (*n* = 57) drawn from 10,620 U.S. respondents. Quantitative responses were compared using T-test & Fisher-Exact tests. Inductive thematic analysis was applied to open-ended questions.

**Results:**

Both U.S. and Chinese samples had relatively high intention to follow preventive behaviors overall. Differences in intended compliance with a specific recommendation appear to be driven by the different cultural norms in U.S. and China. Both groups expressed trepidation about the speed of COVID-19 vaccine development, driven by concern for safety among Chinese respondents, and concern for efficacy among U.S. respondents. The Chinese cohort expressed worries about other countries’ passive handling of the pandemic while the U.S. cohort focused on domestic responses from individuals and government. U.S. participants appeared more knowledgeable on some aspects of COVID-19. Different perspectives regarding COVID-19 origins were identified among the two groups. Participants from both samples reported high trust in health professionals and international health organizations.

**Conclusions:**

Mixed methods data from this cross-national analysis suggests sociocultural differences likely influence perceptions and knowledge of COVID-19 and its related public health policies. Discovering and addressing these culturally-based differences and perceptions are essential to coordinate a global pandemic response.

**Supplementary Information:**

The online version contains supplementary material available at 10.1186/s12992-022-00864-y.

## Background

The COVID-19 pandemic continues to have a devastating global impact [1]. Many COVID-19 mitigation policies are similar across countries. Policy differences are often tied to regional differences in both need and response to the pandemic, such as healthcare capacity, political system, and economic status. Further challenging the global response is that regardless of official policies, pandemic response pertaining to preventive health behaviors depends on the perspectives and reactions of the people themselves. Differences in culturally-based normative behavior may lead to different outcomes under identical policies. To promote a uniform public response, it is therefore important to examine how people living in different regions understand and respond to a pandemic with difference sociocultural backgrounds. This is particularly important for differences between China and the U.S., which have the largest and third largest populations and second and largest economies, respectively (https://databank.worldbank.org). Further, these nations are major financial contributors to the World Health Organization [2], which is often tasked with coordinating global health responses. These commonalities have driven substantial bilateral collaboration in health actions between China and the U.S. [3, 4]; furthermore, the understanding of cultural differences’ impacts on health behaviors supplements the ongoing and unified actions to foster a healthy world.

Considerable differences have been observed in government responses between China and the U.S. Chinese central government has been adhered to its Zero-COVID policy since early pandemic that has involved large-scale lockdowns, mass testing and international travel bans. In contrast, policies in the U.S. have been shifting based on factors including rates of cases and deaths, vaccination rate, and economy. Additionally, public acquisition of COVID-19 information was very different between China and the U.S. In China, COVID-19 information was primarily disseminated by State-run medias (e.g.: *People’s Daily, China Central Television*) on various social network platforms (e.g.: *Weibo*, *Wechat*, and *Douyin*), and unverified information and rumors about COVID-19 were heavily scrutinized and censored [5, 6]. In contrast, misinformation in the U.S. about COVID-19 was rampant on social media, and those who relied on social media had poorer COVID-19 knowledge [7–9]. Remarkably, in spite of these differences, early in the pandemic, adults in both countries reported high intent to comply with public health recommendations [10, 11]. This suggests that cultural differences across different nations may impact individual’s perception and practice towards the COVID-19 pandemic.

When this study was conducted, most global COVID KAP studies were primarily quantitative studies designed to rapidly inform urgent policy decisions [11–13]. Many studies have focused on government responses including the dissemination of COVID-19 public messaging and policies such as public health recommendations; many other studies assessed people’s practices and perceptions regarding the government response and pandemic [14, 15]. While quantitative data provides a wide breadth of knowledge through quantifiable and generalizable insights, qualitative data provides a depth of understanding, and nuance that can help to better understand or explain quantitative findings [16]. Such data helps to understand complex behaviors, perceptions, and attitudes. Unfortunately, there is currently minimal qualitative data in the literature to inform such contextualization and understanding of the potential relationships between people’s practices and their knowledge and perspectives towards COVID-19 pandemic. Such insights are particularly important when considering the role of unique sociocultural differences in cross national studies. To address this gap, this mixed methods study was designed to compare both knowledge and perceptions of COVID-19 between adults in China and a matched cohort in the U.S. through an integration of quantitative and qualitative responses to provide insight into challenges these nations may face in coordinating pandemic response.

## Methods

### Overview

A convergent, mixed methods cross sectional online survey was distributed globally and promoted via snowball recruitment through social media and messaging platforms [16]. The survey examined perceptions about the COVID-19 pandemic. Detailed methods of survey design are described elsewhere [17]. Quantitative and qualitative analyses were conducted separately for Chinese and U.S. respondents [18], and findings compared to draw conclusion. This study was approved by the Penn State College of Medicine Institutional Review Board. All respondents provided implied consent to participate via selection of ‘yes’ to participating in research after reviewing the summary explanation of research. Only participants who select ‘yes’ may advance forward in the survey. Implied consent language, as well as survey question language were translated from original written English form by an IRB-compliant translator into Chinese.

### Survey instrument

A U.S. research team designed the survey, which is provided in an additional file (see Additional file 1 Survey Instrument International), and pilot tested it in central Pennsylvania, described elsewhere [19]. In an effort to rapidly provide usable public health data to inform policy decisions during this medical crisis, we did not formally measure content validation, or formally measure survey reliability. Instead, we completed face and content validity testing using two rounds of cognitive interviewing procedures using the ‘think-aloud’ technique with 13 individuals, followed with pilot testing with a random sample of 1,000 potential participants [20, 21]. The refined survey was then completed by 5,948 individuals. Prior to translation, the survey was again refined based on results, optimized for knowledge discrimination and qualitative sensibility for a global audience. At each of these steps, survey results were evaluated by content experts, and modifications made to each iteration of the survey to optimize content. This iterative development also mirrored a test–retest strategy for confirming reliability; although we did not do that formally, hence, have no Cohen’s kappa for agreement, we can confirm that categorical responses were substantially similar between respondents to the iterative studies and the results of this study.

This process was completed in partnership with the College of Health Information and Management Executives (CHIME®), and included abbreviating the survey and translating it into 23 languages, including Simplified Chinese. An additional tabular data file shows both English and Chinese version of the survey for comparison (see Additional file 2 Chinese survey translation). Translation was completed by two persons fluent in both English and Mandarin. Their translation directive was not literal translation, but rather, interpretive translation to best capture the meaning of English expressions and concepts in the technical language and context of common usage of Mandarin. The final survey used for this project, therefore, had completed face and content validity with thousands of US respondents and culturally sensitive interpretive validation by Mandarin interpreters. The survey was distributed by snowball methods and was administrated online between April 9 and July 12, 2020. An additional movie file shows the survey promotion in Mandarin (see Additional file 3 Chinese survey promotion). Survey distribution was done using email, social media (including YouTube video platform), and press releases by Penn State and CHIME. Respondents provided their responses via an online survey platform, Surveyhero [22].

### Measures

Conceptual design of the survey was based off the European ‘Standard questionnaire on risk perception of an infectious disease outbreak’ with qualitative additions in the form of open-ended questions added to allow for a mixed methods study design [23]. Demographic information collected included age, sex, education level, and perceived social standing [24]. Qualitative analysis was based on free text responses to five open-ended questions: 1) ‘*What prevents you from following these recommendations more often?’;* 2) *‘In what way has the COVID-19 pandemic changed the way you consume news?’;* 3) *‘How do you feel about reopening?’;* 4) *‘What are your thoughts about a potential COVID-19 vaccine?’;* and 5) *‘What is your understanding of where and how COVID-19 started?’* The quantitative portion of the survey was organized into 5 constructs, detailed below.

#### Construct 1

*Intent to comply with public health recommendations*. Eight Likert-scaled items were originally included in the survey to assess an intent score (range: 1–5; 5 = will always follow the measure). In addition, average scores of items were calculated to indicate an overall level of compliance for each respondent.

#### Construct 2

*Information consumption during the pandemic.* One item, a dichotomous question (Yes/No), assessed whether participants changed their news consumptions during the pandemic. A single-select multiple choice question was also used to gather participants’ major news sources during the pandemic.

#### Construct 3

*Intent to receive a COVID-19 vaccine.* Four Likert-scaled items (range: 1–5; 5 = highest intent) were included. An average score of 4 items was calculated to describe each participant’s overall tendency level towards vaccinations.

#### Construct 4

*Trust in common information sources.* Six Likert-scaled items (range: 1–5; 5 = complete trust) were used to measure trust towards each of six different sources, including World Health Organization, primary care provider, national and local governments, and CDC.

#### Construct 5

*Overall knowledge related to COVID-19*. Seven dichotomous items (True/False) were used. The proportion of corrected responses between the samples was compared per question, as was the mean difference between respondents’ overall knowledge.

### Cohort matching and sampling

There were 11,920 survey respondents from the single global survey. Of those, 10,620 lived in the US, and 57 respondents were from China. The remainder of the participants were from other countries. This discrepancy in sample size was observed primarily due to a lack of exposure from foreign media platforms in China. To address this sampling discrepancy and selection bias, a U.S. cohort (*n* = 57) was identified by iteratively matching every Chinese respondent for sex, age (within five years), survey completion date (within five days), education level, and perceived social standing, in that order. From each matched pool, a single respondent was randomly chosen. After the match, one Chinese respondent was determined to be a German citizen transiently living in China, and was excluded for final cohort sizes of 56 from China and 57 in the matched U.S. cohort. Match criteria were selected as factors associated with differences in COVID-19 knowledge and perceptions [19, 25, 26] and to avoid information saturation bias.

### Qualitative analysis

We used an ontological philosophical assumption that views reality as seen through multiple views [27]. Further, we used a pragmatic approach that appreciates the diversity of contexts in which the research occurred [27]. We applied descriptive thematic analysis in order to understand individuals’ common, lived experiences [28]. Two independent teams were formed: one to analyze the U.S. cohort data (*n* = 57) and one to analyze the Chinese cohort data (*n* = 56) to maintain independence of analysis. Each team included both English and Chinese speaking analysts, and used the same process of thematic analysis [28].

NVivo version 12.0 was used for qualitative analyses. An inductive process was used to develop codes that emerged after review of all free-text survey responses. The Chinese free-text responses were analyzed in native format without translation. Consensus on codebook definitions was achieved through group discussions. The constant comparison method was used to code data [29]. The preliminary codebook was used to code approximately 20% of survey responses. Coding discrepancies were reconciled through discussion and grounding in source data. Intra-class reliability was measured by Cohen’s kappa, which was greater than 0.7 for the U.S. and Chinese datasets respectively and separately [30].

After coding was completed, each analysis team independently conducted a content analysis of their coded data. Then, the US and Chinese coding teams convened to share content-level qualitative findings across all 5 questions, after which a more in-depth overarching thematic analysis was conducted to establish an integrated narrative across both country datasets [28]. Similarities and differences in themes that emerged between the U.S. and Chinese samples were explored using the constant comparison method [31]. Biases were bracketed by grounding the analysis in verbatim quotations to maintain neutrality and credibility of the themes [32, 33].

### Quantitative analysis

Demographic survey items were characterized using descriptive statistics. For dichotomous questions, Fisher’s exact tests were utilized to examine the frequency differences; For Likert-scale questions, parametric and non-parametric tests were used to compare normally and non-normally distributed means, respectively. Statistical significance level was set at *p* < 0.05 (two sided). Statistical analyses were conducted using R statistical software [34].

### Mixed methods integration

Quantitative and qualitative findings from both samples were integrated using a narrative approach to assimilate quantitative and qualitative findings between samples [35]. We use ‘weaving’ to present our findings [35], which involves presenting our results through a mixed methods joint display table (Table 1, shown at the beginning of Results section). Of which, major themes we deduced from the qualitative analyses based on the open-ended questions in the survey were summarized, then we supplemented quantitative analyses before describing the coherence or discrepancies from both qualitative and quantitative data.

**Table 1:** Joint display of perceptions and knowledge regarding the COVID-19 pandemic between China and U.S. samples

Qualitative Themes	Qualitative Findings	Quantitative Survey Items	Quantitative Findings			Mixed Methods Interpretation
			Cohort (N)	Mean/Proportion	p-value*	
**Barriers to adhering to recommendations**	**China:** Participants acknowledged recommendations but cited difficulty following them due to restrictions in the physical environment, unconscious habits, demands of daily life	**Intent to comply score** **(Range: 1–5)**	**China** **(*N* = 55)**	Mean = 4.19 (SD = 0.6)	** < 0.01**	While both countries’ intent to follow recommendations was high based on the quantitative analyses, the U.S. intent was higher Qualitative analyses identified cultural differences in response to cough etiquette recommendations as the primary cause of the difference seen
	**U.S.:** Participants reported following recommendations themselves but noted that others around them did not follow public health recommendations		**U.S** **(*N* = 57)**	Mean = 4.46 (SD = 0.47)		
**Thoughts and comments on COVID-19 vaccine**	**China:** Participants express a ‘safety-first’ mentality towards vaccine development	**Overall tendency towards COVID-19 vaccination (Range: 1–5)**	**China** **(*N* = 22)**	Mean = 4.25 (SD = 0.36)	0.343	Based on quantitative results, both groups showed very supportive attitudes towards vaccination Differences driving skepticism was found in qualitative responses
	**U.S.:** Participants express concern over efficacy of the vaccine due to the fast-track development and deployment		**U.S** **(*N* = 21)**	Mean = 4.43 (SD = 0.79)		
**Perspectives on reopening**	**China:** Participants report a more global perspective to reopening, citing ‘we’ will all be ‘ok’ if others do better	**Trust score towards national/federal government** **(Range: 1–5)**	**China** **(*N* = 56)**	Mean = 4.3 (SD = 0.89)	** < 0.001**	Quantitative and qualitative responses indicated different outbreak stages two groups were experiencing might have impacted their perceptions towards reopening. By the time of survey, the outbreak was under control in China while U.S. started to surge. U.S. participants had lower overall trust towards the government than their Chinese counterparts, although both samples challenged the timing of reopening
			**U. S** **(*N* = 57)**	Mean = 2.39 (SD = 1.1)		
	**U.S.:** Participants express widespread concern that the U.S. is reopening too soon and cite the need for a comprehensive plan for reopening from the government with strong political undertone	**Trust score towards local government** **(Range: 1–5)**	**China** **(*N* = 54)**	Mean = 4.17 (SD = 1)	** < 0.001**	
			**U. S** **(*N* = 57)**	Mean = 3.53 (SD = 0.97)		
**Change of COVID-19 messaging consumptions**	**China:** Diversifying information sources and turning to new information tools	**Have changed ways of consuming COVID-19 related news** **(Yes/No)**	**China** **(*N* = 56)**	19%	** < 0.001**	Quantitatively, more U.S. participants changed their news consumption because of COVID. Both groups relied primarily on internet sources Qualitatively, both groups expressed seeking news from a broader array of sources to determine ‘real’ news
	**U.S.:** Turning to new information sources, being more cautious about information and consuming new more now than prior to pandemic		**U.S** **(*N* = 57)**	54%		
**COVID-19 pandemic related information**	**China:** Participants were ambiguous in identifying the origins of the COVID-19 virus. Some demonstrated unclear identification of viral origins and/or index case(s)	**Overall COVID-19 knowledge Score** **(Range: 0–7)**	**China** **(*N* = 34)**	Mean = 4.68 (SD = 1.55)	**0.021**	Both quantitative and qualitative analyses suggested COVID-19 pandemic related information was not clear to all respondents Differences were most robust with regards to the origin of virus. This may be related to perceptions rather than knowledge, particularly in the context of the evolving knowledge about the virus
	**U.S.:** Participants objectively reported their perceptions of COVID-19 pandemic origins- geographically as beginning in Wuhan, China with likely zoonotic transmission		**U. S** **(*N* = 24)**	Mean = 5.42 (SD = 0.78)		

^*^Two-tailed Fisher exact test to 95% confidence. **Bold** values are significant

## Results

Demographics for each cohort are shown in the Table 2 below. Participants were mostly young adults (ages 18–34, 61.1%), who were well educated (bachelor’s or higher degree, 90.2%), with above average self-identified social standing (6.13 out of 10).

**Table 2:** Demographic characteristics of the study participants in China and U.S

Demographic Characteristics		Chinese Cohort (*n* = 56)	U.S. Cohort (*n* = 57)	p*
**Age**	Mean	34.7 (SD = 9.26)	35.5 (SD = 11.11)	0.678
	Percent by age group			
	18–24	0.0%	3.5%	0.690
	25–34	64.3%	57.9%	
	35–44	19.6%	21.1%	
	> 44	16.1%	15.8%	
**Sex**	Male	51.8%	47.4%	0.904
	Female	42.9%	47.4%	
	Prefer not to answer	5.3%	5.2%	
**SES Ladder**	Mean Score	6.14 (SD = 1.43)	6.12 (SD = 1.45)	0.941
**Education Attainment**	High School	1.8%	0.0%	0.919
	Associate Degree	8.9%	8.8%	
	Bachelor	32.1%	36.8%	
	Graduate	57.1%	54.4%	

^*^T-tests & Fisher’s exact tests, 2-sided, to 95% confidence. No difference is significant

### Barriers to following health recommendations

While quantitatively, most participants from both countries were willing to follow COVID-19 related health recommendations, different barriers were identified in qualitative analysis.

#### Qualitative findings

Some Chinese respondents mentioned limited physical space in public areas, *‘交通工具上难以保持距离. (It is hard to keep the distance on public transportation.)’* Further, individuals noted that changing social behaviors was challenging, e.g., *‘以前的生活, 社交习惯 (Hard to change lifestyle and social habits)’* particularly since they did not feel their safety was imminently threatened. A Chinese participant (28 years old, male, bachelor’s degree, Jiangxi) wrote:

*‘如果是疫情最严重的时候自然会遵守, 甚至不会出门, 但现在中国江西几乎没有了, 所以便不会那么注意. (It's natural to follow the rules when the pandemic was at the worst situation, I wouldn't even go out of my home. But currently Jiangxi barely has cases, so I am not being so careful now.)’*

Meanwhile, U.S. participants reported their biggest concern was others not following public health recommendations. One stated (35 years old, female, Massachusetts), *‘I am worried about people not taking it seriously or not being as careful, especially as the weather gets warmer. I am also worried about people becoming over-confident and cavalier.’*

#### Quantitative findings

Due to a low number of responses received for the last three items (48.7% missingness for each item), the mean score for each respondent was only aggregated based on the first five survey items. U.S. participants had higher aggregate compliance intent. However, this aggregate difference was driven primarily by a marked difference in item 4, cough etiquette. Results from item-wise comparison are shown in the Table 3 below.

**Table 3:** Comparison of intent to comply by question

Question	China (*n* = 55)		U.S. (*n* = 57)		p^*^
	**Mean**	**SD**	**Mean**	**SD**	
1. Wash your hands often (for 20 s or more)	4.27	0.78	4.47	0.68	0.150
2. Wear a cloth face cover (facemask) when out in public	4.18	0.82	4.44	0.8	0.096
3. Avoid touching your eyes, nose, and mouth with unwashed hands	3.98	0.95	4.05	0.91	0.689
4. Cover your mouth and nose with a tissue or the inside of your elbow when you cough or sneeze	3.93	1.2	4.58	0.71	** < 0.001**
5. Stay home if you feel unwell	4.56	0.6	4.77	0.5	**0.049**
Total	4.19	0.6	4.46	0.47	**0.007**

^*^T-test used, 2-sided, to 95% confidence. **Bold** values are significant

### Skepticism about the COVID-19 vaccine

Both groups indicated a willingness to take a COVID-19 vaccine, but also noted skepticism stemming from the vaccine’s rapid development. Interestingly, their skepticism led to different concerns.

#### Qualitative findings

Chinese participants expected that a COVID-19 vaccine would eventually be successfully developed but were concerned that rapid development might compromise the vaccine’s safety. One respondent (38 years old, male, graduate degree) wrote,

‘希望在保证安全和质量的前提下尽快研制成功. (Hope vaccines can be successfully developed as soon as possible with the assurance of safety and quality.)’

In contrast, U.S. participants focused primarily on concern that rapid development might compromise vaccine efficacy. One participant (25 years old, female, graduate degree, Illinois) said, *‘I want there to be a vaccine, but worry that it is so fast tracked that it could have side effects or be ineffective*.’

#### Quantitative findings

A reduced sample size was observed for these four items given the fact that disclosing personal vaccination preferences might be a sensitive topic for participants from both countries. Both groups indicated high overall tendency towards COVID-19 vaccination, with no significant difference between them (China: *n* = 22, Mean = 4.25, SD = 0.36; U.S.: *n* = 21, Mean = 4.43, SD = 0.79; *p* = 0.343).

### Perspectives on reopening

At the time of survey (April-July 2020), reopening of the U.S. was under consideration in many states [36]. In contrast, China just lifted its lockdown on Wuhan city, and people started to follow more global news on the pandemic. Neither cohort supported reopening.

#### Qualitative findings

Chinese respondents’ expressed concerns about the global impact of reopening – particularly driving war and international discontent. One participant (39 years old, female, graduate degree) wrote,

*‘死亡, 治愈后遗症, 次生灾难, 如经济衰退, 失业, 战争, 心理疾病等. (Deaths, post covid19 syndrome, second-order damages such as economic recession, unemployment, war, and mental disorders).’*

Another (44 years old, graduate degree) worried that reopening would lead to, *‘世界互相指责和分裂. (a blame game that makes the world divided)’*, and some (30 years old, male, associate degree) worried that uncoordinated reopening would undermine success, noting,

‘*在其他国家已经控制住疫情漫延的情况下, 很多国家放弃控制, 那么之前的所有努力都会付诸东流. (In the situation where the outbreak is under control in some countries while many other countries give up responding to COVID-19, all the efforts done previously will come to naught).’*

The U.S. cohort primarily considered reopening within their country, with many expressing concerns that premature reopening without a robust protective strategy would be disastrous. One U.S. participant (28, female, bachelor’s degree, Illinois) said:

*‘Am very stressed about it. I feel like we all know how this ends. History and science have told us these things. Unfortunately, it seems that our country has chosen profit over people. The whole world is watching as our curve never flattens, and we prepare for what is likely another wave later in the year.’*

#### Quantitative findings

With regards to trust in government, Chinese participants reported higher trust compared to the U.S. cohort, both at a national (China: *n* = 56, Mean = 4.30, SD = 0.89; U.S.: *n* = 57, Mean = 2.39, SD = 1.1; *p* < 0.001) and local level (China: *n* = 54, Mean = 4.17, SD = 1; U.S.: *n* = 57, Mean = 3.53, SD = 0.97; *p* < 0.001).

### Change of COVID-19-related information consumption

Both samples reported that the internet was their primary information source and that their change in news consumption was based on a perceived need to diversify information sources to get the ‘real’ news.

#### Qualitative findings

Both samples noted that distrust of information led them to increase and diversify information sources for improved accuracy (i.e., more use of government tracking sites, *‘疫情图 (COVID-19 tracking map)’*, and social media, *‘社交媒体 (social media)’*. Participants also attempted to identify more personally relevant information. For example, a U.S. participant (30, female, bachelor’s degree) wrote,

*‘I started paying attention more closely to my local newspapers because I needed to stay up to date on local and state ordinances - learning which businesses would remain open, what reopening meant, case numbers in my area, whether testing was available, and so on. Also, I started paying more attention to more scientific journals or science-focused publishing, which had less of a political focus.’*

#### Quantitative findings

**Fig. 1:**
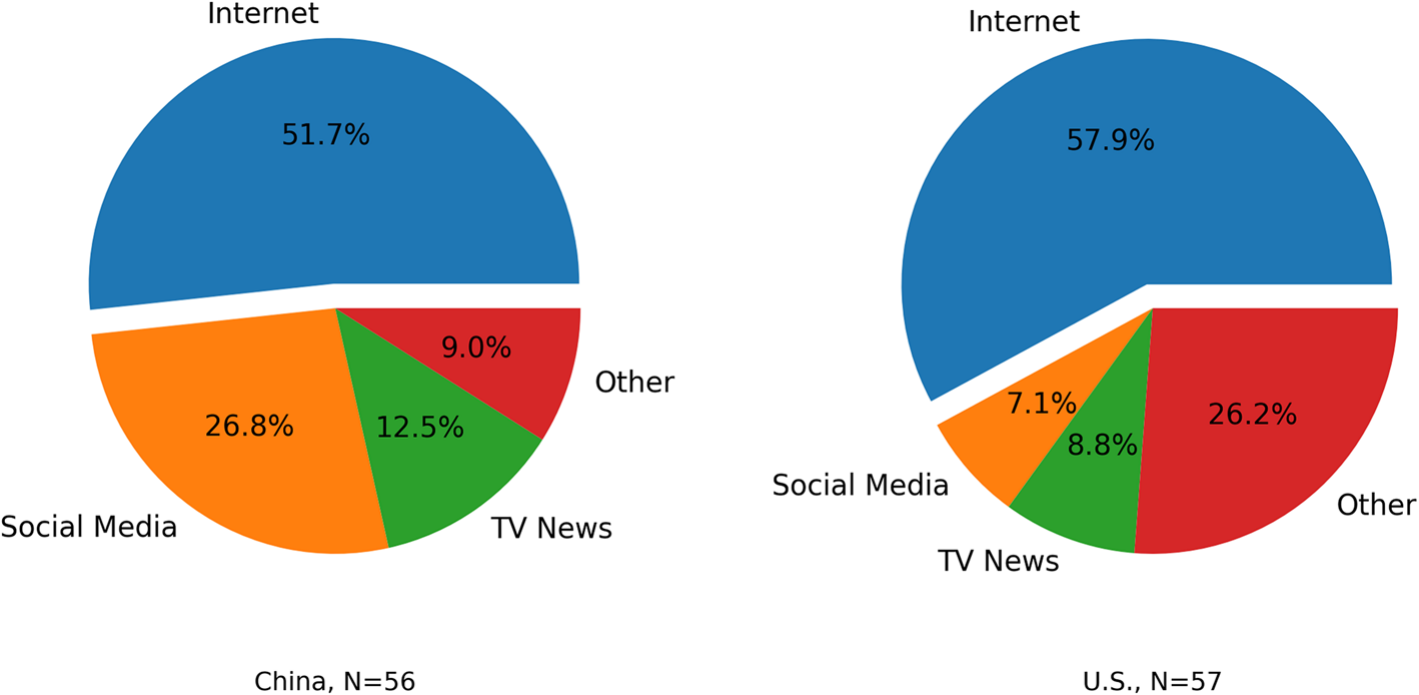
COVID-19 news sources between China and U.S. samples, April- July 2020

U.S. respondents were more likely to report that they had changed their consumption of news because of COVID-19 (54% versus 19%, *p* < 0.001). Over half of both samples got their health-related news from news websites, government websites, and other internet sites (social media excluded, see Fig. 1 below). Both samples showed similar high trust in health professionals, including personal health providers (China: *n* = 48, Mean = 4.08, SD = 1.07; U.S.: *n* = 54, Mean = 4.2, SD = 0.66; *p* = 0.501) and WHO (China: *n* = 55, Mean = 4.13, SD = 1.04; U.S.: *n* = 56, Mean = 3.96, SD = 1.01; *p* = 0.403).

### COVID-19 pandemic related information

Qualitative analysis showed differences in perceptions about the origins of COVID-19. U.S. respondents answered more questions correctly than the Chinese cohort.

#### Qualitative findings

In the free-text responses about the origin of COVID-19, answers from the Chinese cohort were vague and indirect. One response (38 years old, male, graduate degree) wrote,

*‘病毒溯源是非常艰难的科学问题, 该病毒有可能是多个源头几乎同时爆发, 中间宿主也尚不明确 (Tracing the source of virus is a difficult scientific problem. SARS-CoV-2 might originate from and cause the outbreak at multiple places simultaneously. Intermediate hosts for the virus are still undetermined.)’*

In contrast, U.S. respondents were clear in their free-text responses about the origin of COVID-19, using key words like *‘Wuhan, China’*, *‘wet market’*, and *‘zoonotic transmission.’*

#### Quantitative findings

U.S. respondents answered more of the seven knowledge questions correctly (range: 0–7, 7 = all questions correctly answered; China: *n* = 34, Mean = 4.68, SD = 1.55; U.S.: *n* = 24, Mean = 5.42, SD = 0.78; *p* = 0.021). This difference appears primarily driven by Question 7, with no significant difference between groups on answers to the other questions (See Table 4 below). Remarkably, more than 2/3 of participants from both groups did not know that the public health recommendations from WHO, European Commission, and U.S. CDC were different.

**Table 4:** Comparison of COVID-19 knowledge by question

Question (*Answer*^*a*^)	Correct Responses (%)		*p*^†^
	**China (*n* = 34)**	**U.S. (*n* = 24)**	
1. When gathering with others, it is safer (from COVID-19) to meet indoors than outdoors (*FALSE)*	85.3%	100%	0.070
2. A person with COVID-19 can infect other people even if they have no symptoms of COVID-19 (*TRUE*)	94.1%	100%	0.506
3. A vaccine for COVID-19 is available in some countries (*FALSE*)	82.4%	83.3%	0.999
4. The World Health Organization, European Commission, and U.S. Centers for Disease Control and Prevention all have the **same** public health recommendations to reduce the spread of COVID-19 (*FALSE*)	32.4%	29.2%	0.999
5. Treatments for mild symptoms of COVID-19 are available without a prescription (*TRUE*)	41.2%	50%	0.596
6. A positive antibody test for COVID-19 determines **when** you contracted the disease (*FALSE*)	61.8%	83.3%	0.089
7. Most people who get COVID-19 will survive (*TRUE*)	70.6%	95.8%	**0.019**
Overall	4.68	5.42	**0.021**

^a^Answers were true based on information publicly available on the U.S. CDC and/or WHO websites at the time of the survey

^†^Two-tailed Fisher exact test to 95% confidence. **Bold** values are significant

## Discussion

These results highlight differences in knowledge, thoughts, and perspectives about COVID-19 and vaccination between China and the U.S early in the pandemic. These data offer insight into cultural differences that may drive differences in behavior and information processing. Thematically, the Chinese responses focused on more global and community health concerns than the U.S. responses that focused more on domestic response and fears related to the behaviors of others.

Understanding these culture differences is important when analyzing cross-national data, particularly in aggregate. Consideration of these differences may aid in interpretation. For example, while differences in intent to comply with recommendations are seen, they are most pronounced with regard to ‘cough etiquette’, with significant cultural and social mediators. Cough etiquette has been well established in the U.S. for many years and absorbed into culture [37], yet is a relatively new recommendation in China, so it is not surprising that Chinese participants indicated lower intent to comply with that recommendation.

Differences in social and cultural norms also appear to influence COVID-19 vaccine concerns. While both samples were accepting of a vaccine in spite of their concerns about rapid development, U.S. respondents were concerned about *efficacy*, while Chinese respondents were worried about *safety*. While that may have been influenced by a domestic (Chinese) vaccine scandal reported around the time of the study [38, 39], the difference highlights the need to tailor vaccination campaigns based on these culture differences.

**Fig. 2:**
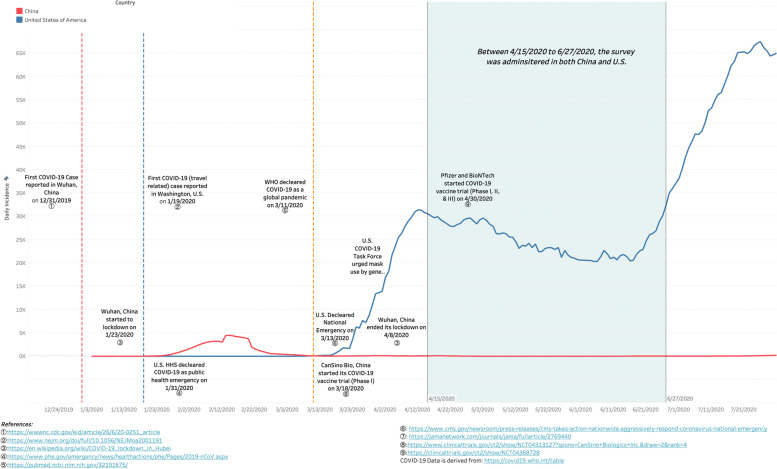
COVID-19 incidence curve between China and U.S., December 2019- July 2020

The overtly political undertone among U.S. responses was absent among Chinese responses, and may have been due to differences in where each country was on the epidemic curves (shown in Fig. 2 below) when survey responses were collected. The spread of COVID-19 had slowed in China while it was surging in the U.S., with high levels of public dissatisfaction and criticism of the U.S. government’s response [40]. In contrast, with their first wave waning, Chinese participants were observing as COVID-19 ravaged other countries, which may have inspired their global perspective. Chinese respondents considered SARS-CoV-2 to be a deadlier threat than U.S. respondents, which may also have stemmed from being on different points on the pandemic curve. At the time of the survey, China had higher confirmed cases and deaths, and a contingent of the U.S. considered the pandemic to be a ‘hoax’ [41]. This perception from Chinese participants may have not only increased empathy to other countries, but sparked concern that failure of other countries to contain the pandemic would expose China to a second wave of infection.

Individualism versus collectivism may partly explain the differences in trust in government [42]. U.S. respondents, who look to individuals to solve problems and are more critical of government policies, had low trust in government, while Chinese respondents, who look to the government as a voice of the collective and are more accepting of government measures, had high trust in government. This may also influence attitudes towards mitigation behaviors. The U.S. values individualism, hence, assumes self-reliance, with a primary obstacle being interference by others. This is reflected in U.S. respondents’ concern about others’ failure to comply with public health recommendations. China values collectivism, hence, focuses on the needs of the community, and are more dependent on the community in return. For example, pre-COVID-19, more than half of Chinese consumers made 2–3 trips to the grocery store per week, while U.S. consumers averaged only 1.5 trips [43, 44]. The rapid development of community group buying activities in China in which a designated community leader, normally a government official, coordinates large food orders on behalf of a group of people – especially those at higher risk from COVID-19 [45] is evidence that community-oriented response influenced other COVID-19 mitigation behaviors.

Information dissemination is another crucial component in controlling the pandemic [9, 46]. Comparison of the data between participants from two countries suggests an information gap, with the U.S. cohort absorbing more detailed COVID-19 information. Media differences between these countries include platforms used, method of dissemination, strategies of fact-checking, and (government) information scrutinizing. Our data suggests that while U.S. participants had increased news consumption and were more knowledgeable related to COVID-19, the diversity of news outlets sometimes produced inconsistent information contrary to best health practices. Chinese responses suggest that the media could serve a more active role to ensure increased awareness of COVID-19 related information. It is concerning that respondents’ search for diverse information sources included social media, as those using social media as a COVID-19 source have been shown to have lower COVID-19 knowledge [47].

This is, to our knowledge, the first mixed methods comparison of COVID-19 knowledge and perspectives between China and the U.S. Strengths of our study include the robust U.S. response, which enabled nearly perfect case matching to the Chinese respondents. Also, our bilingual analytic team avoided content and sentiment loss through translation. Another strength is that the survey itself was a refinement of a survey previously developed, improving qualitative sensibility.

The primary limitation of this study is the mismatch in response rate between Chinese and U.S. respondents. However, this sample size limitation is addressed by a near-perfect match to a U.S. cohort. Our study also is limited by selection bias across both samples based on those amenable to participation in research and those who have access to online platforms for survey completion. Further, those who are less active on online platforms might respond differently than participants in this analysis [48]. Internet communities between two countries also differed considerably (with information dissemination being part of the differences), and such differences might be imbedded into sociocultural elements which will need to be further investigated. Additionally, Chinese respondents were primarily highly educated, from metropolitan areas, and few in number compared to the population of China; hence, with the cohort matching strategy, themes from both samples may not be generalizable to other regions and populations in two countries.

Our results are also limited by a lack of formal content validity and reliability testing—necessary to obtain data in time to be of use early in the pandemic. These limitations are mitigated by starting with the Standard European survey, and through iterative improvements informed by participant results and expert review, as well as through response similarity by demographic across survey iterations. As a cross-sectional survey, results may not be generalizable over time, and it was not possible to conduct follow-up interviews to clarify points of question or confusion. Since questions were developed by U.S. researchers who did not speak Chinese, differences in interpretation may have occurred. To address this, we included two bilingual qualitative researchers who deployed various means (i.e.: review open-ended questions before coding, cross-validation of the qualitative results, and rigorous statistical tests) to clean the quantitative and qualitative data for synthesis. Even so, some qualitative questions may not have had the same sensibility, to Chinese respondents. For example, Chinese participants may have interpreted ‘where COVID-19 started’ as an open philosophical or detailed biological question, hence, may have been more likely to state the ‘correct’ answer, ‘Wuhan, China,’ if they had been asked, ‘where was COVID-19 first reported.’ However, survey translation was completed by fluent Chinese speakers and performed with a goal of interpretation, conveying accurate meaning behind quantitative and qualitative questions instead of word substitution. Last, it is also important to emphasize that the cross-national differences in culture and social backgrounds across the U.S. and China. These include but are not limited to potential differences in pandemic experiences and timing in the US and China, differences in information dissemination, national response plans, and other societal factors that may have affected participants’ experiences and survey responses. In future studies, research that is closely aligned with local informants with attention to regional timeline variations could help assess these issues.

## Conclusions

Culturally driven differences in COVID-19 knowledge, perceptions, preferred information sources, and intent to comply with public health recommendations between countries challenge the call for a unified, global response to COVID-19 [49]. What works for one country might not work for another. Understanding these differences in cultural and social norms is essential to global cooperation; identifying cross-culture similarities reveals bridges we can use to facilitate overcoming our differences. Given the turmoil of this pandemic, perhaps the most important result of this study is that despite the differences we identify, among respondents with two very different cultures, languages, and governments, we see similarities in understanding the severity of threat of COVID-19, the importance of complying with public health recommendations, and a high regard for health professionals and international health organizations. Whatever our differences, our results reveal a shared humanity that may be leveraged to better coordinate global health responses.

## Supplementary Information

**Additional file 1. **Survey instrument international. Communicating with patients during the COVID-19 pandemic. The original international survey was developed by Penn State and CHIME and was available online between April 9 and July 12, 2020.

**Additional file 2. **Chinese survey translation. Survey translation. The original survey in English and its translated version in Chinese.

**Additional file 3. **Chinese survey promotion. Survey promotion in Mandarin. Survey promotion in Mandarin when distributed online using snowball method.

## Data Availability

Both qualitative and quantitative data was available upon reasonable request by contacting Robert Lennon (rlennon@pennstatehealth.psu.edu).
