# Supplementary material for: Perceptions and knowledge regarding the COVID-19 pandemic between U.S. and China: a mixed methods study
Source: Global Health. 2022 Aug 8;18:76. doi: 10.1186/s12992-022-00864-y (PMC9358088; doi:10.1186/s12992-022-00864-y)
Supplement: Supplementary file 1 — Additional file 1. Survey instrument international. Communicating with patients during the COVID-19 pandemic. The original international survey was developed by Penn State and CHIME and was available online between April 9 and July 12, 2020. [file 12992_2022_864_MOESM1_ESM.pdf]

Complete translation available on reasonable request.

Communicating with Patients During The COVID-19 Pandemic

Good day,

The world's ability to respond to the pandemic of novel coronavirus 2019 (COVID-19) depends on effective communication so that all people understand what they can do to protect themselves and their communities.

We would appreciate your help by participating in a research survey to help us understand your opinion about preventive measures to protect yourself and your communities from COVID-19.

We would also like to record your experiences of this event so that people today and in the future can learn from them. There are several places in the survey for you to record as much or as little of your story as you like in your own words.

Your individual responses to this survey will remain anonymous! No personal identifying information is recorded, and no individual survey information will be transmitted to any government or health authority.

Please allow approximately 10 minutes to complete this survey. We would be grateful if you would complete this survey by 11:59 pm, Eastern Standard Time, June 30, 2020.

Are you 18 years old or older?	○Yes<br>○No

SUMMARY EXPLANATION OF RESEARCH

Penn State College of Medicine
Penn State Health
Title of Project: COVID-19 Health Messaging Efficacy and its Impact on Public Perception, Anxiety, and
Behavior
Principal Investigator: Robert P. Lennon, MD, JD, FAAFP
Address: 500 University Drive
PO Box 850, MC HS72
Hershey, PA 17033-0850

You are being invited to volunteer to participate in a research study. Research studies include only people who voluntarily choose to take part. This summary explains key information about this research.

You are urged to ask questions about anything that is unclear to you.

- **The purpose of this study is to understand how messaging during the COVID pandemic is being received by the general population.**

- **You are being asked to take a survey. You are free to skip any questions you do not wish to answer in the survey.**

- **It is expected that it will take 10 minutes to complete the survey**

- **You may experience slight discomfort or anxiety as you complete the survey. The confidentiality of your electronic data created by you or by the researchers will be maintained to the degree permitted by the technology used. Absolute confidentiality cannot be guaranteed; however, no personally identifying information will be collected.**

- **There is no direct benefit of you participating in this study, however, the results of this research have timely implications on guiding on nation's policymakers and health leaders on how to better message during the COVID pandemic, and future public health emergencies.**

- **There is no compensation provided for participating in the survey.**

- **Funding for this research study is provided by the Huck Institutes of the Life Sciences and the Social Science Research Institute at Penn State University as well as the Department of Family & Community Medicine at the Penn State College of Medicine.**

- **No identifiable health information will be collected in this survey.**

- **Your permission for the use and sharing of your identifiable health information will continue indefinitely.**

- **In the event of any publication or presentation resulting from the research, no personally identifiable information will be shared (as none is collected). You have the right to ask any questions you may have about this research.**

If you have questions, complaints or concerns or believe you may have been harmed from participating in this research, you should contact Dr. Lennon at myCOVIDstory@psu.edu. If you have questions regarding your rights as a research subject or concerns regarding your privacy, you may contact the research protection advocate in the HMC Human Subjects Protection Office at 717-531-5687. You may call this number to discuss any problems, concerns or questions; get information or offer input.

In Wisconsin, you may also contact Linda N. Meurer, MD, Medical College of Wisconsin, at lmeurer@mcw.edu or 414-955-5724 about the survey. If you have questions about your rights as a research participant or want to report any problems or complaints, you can call the Medical College of Wisconsin/Froedtert Hospital Research Subject Advocate at (414) 955-8844.

You do not have to participate in this research. Taking part in the research study is voluntary. Your decision to participate or to decline the research will not result in any penalty or loss of benefits to which you are entitled.

Your completion of the questionnaire implies your voluntary consent to participate in the research and to allow your information to be used and shared as described above.

Do you wish to proceed to the survey?<br><br>By selecting 'yes' you are agreeing to participate in this study.	○Yes<br>○No

SECTION 1. Public Health Recommendations

How often do you currently follow these public health recommendations?

	Never	Rarely	Sometimes	Most of the time	Always
Wash your hands often with soap and water for at least 20 seconds	○	○	○	○	○
Wear a cloth face cover (facemask) when out in public	○	○	○	○	○
Avoid touching your eyes, nose, and mouth with unwashed hands	○	○	○	○	○
Cover your mouth and nose with a tissue when you cough or sneeze or use the inside of your elbow	○	○	○	○	○
Stay home if you feel unwell.	○	○	○	○	○
If you have a fever, cough, and difficulty breathing seek medical attention and call in advance.	○	○	○	○	○
Stay at least 6 feet (2 meters, or about 2 arms' lengths) from other people when outside of your home	○	○	○	○	○
Stay out of crowded places and avoid mass gatherings	○	○	○	○	○

{Branching Logic} IF above has any answer of “Never, Rarely, or Sometimes”
What prevents you from following these recommendations more frequently? {freetext}

Please be as detailed as possible in your response.<br>You may write as much or as little as you want.<br>There is no word limit.	____

SECTION 2. How do you feel about reopening?
(Re-opening means lifting government quarantines/restrictions to prevent the spread of COVID-19)

Please be as detailed as possible in your response.<br>You may write as much or as little as you want.<br>There is no word limit.	____________________________________

SECTION 3. Information preferences

Has the COVID-19 pandemic changed the way you consume news?	○Yes<br>○No
{If yes}<br>In what way has the COVID-19 pandemic changed the way you consume news?	

From where do you consume most of your health information?	Facebook®<br>Family & Friends<br>Government officials<br>Instagram®<br>Internet: Government Websites (WHO, CDC, EU)<br>Internet: News Websites<br>My faith leaders<br>My healthcare provider<br>Podcasts<br>Print News<br>Radio<br>Television News channels<br>Twitter®<br>Other Internet Sites<br>Other Social Media<br>Other
Please type in your single most used source. For example, "XYZ News.com"	

Where do you turn when you really want to know if health information is true?<br>Select all that apply.	Facebook®<br>Family & Friends<br>Government officials<br>Instagram®<br>Internet: Government Websites (WHO, CDC, EU)<br>Internet: News Websites<br>My faith leaders<br>My Healthcare Provider<br>Podcasts<br>Print News<br>Radio<br>Television News channels

	Twitter®<br>Other Internet Sites<br>Other Social Media<br>Other
BRANCHING FOLLOW UP TO ABOVE	
If you are comfortable sharing, please type in your religion	
Please type in which internet news source(s)	
Please type in which podcast(s)	
Please type in which print news source(s)	
Please type in which radio source(s)	
Please type in which television news channel(s)	
Please type in which Twitter® feed(s)	
Please type in which other website(s)	
Please type in which other social media source(s)	
Please type in which other source(s)	

SECTION 4. Trust in Vaccines

Has COVID-19 changed your perceptions on vaccination? (Y/N)

What is your understanding of a future COVID-19 vaccine – what have you heard and do you believe it? {freetext}

Please describe how much you agree or disagree with the following statements.

	Strongly disagree	Disagree	Neither agree nor disagree	Agree	Strongly agree
I am likely to get the COVID-19 vaccine when it becomes available.	○	○	○	○	○
My family and friends would support me to get vaccinated for COVID-19.	○	○	○	○	○
A COVID-19 vaccine will be important to protect myself against COVID-19.	○	○	○	○	○
A COVID-19 vaccine will be important to protect the health of my community.	○	○	○	○	○
I trust the current system for evaluating the safety of the COVID-19 vaccine.	○	○	○	○	○

SECTION 5. The extent to which you trust common information sources.

Please rate how much you trust the following information sources.

	Not at all	Not much	A little	Quite a bit	Completely	Not Applicable
Your national (federal) government officials	○	○	○	○	○	○
Your local government officials	○	○	○	○	○	○
Your personal healthcare provider (the person who most frequently provides your care)	○	○	○	○	○	○
The World Health Organization (WHO)	○	○	○	○	○	○
The U.S. Centers for Disease Control and Prevention (U.S. CDC)	○	○	○	○	○	○
The European Commission	○	○	○	○	○	○

SECTION 6. Your understanding of COVID-19

You are not expected to know all the answers.

	True	False	Not Sure
When gathering with others, a person is **less likely** to get COVID-19 inside than outside	○	○	○
A person with COVID-19 can infect other people even if they have no symptoms of COVID-19	○	○	○
A vaccine for COVID is available in some countries	○	○	○
The World Health Organization, European Commission, and U.S. Centers for Disease Control and Prevention all have the **same** public health recommendations to reduce the spread of COVID-19	○	○	○
Treatments for mild symptoms of COVID-19 are available without a prescription	○	○	○
A positive antibody test for COVID-19 determines **when** you contracted the disease	○	○	○
Most people who get COVID-19 will survive	○	○	○

Perception of COVID-19 relative to flu.
How likely is it that you will be diagnosed with flu or COVID-19? (If you have been diagnosed with any of these in the past three months please select that answer.)

	Very unlikely	Unlikely	Possibly	Likely	Very Likely	I have been diagnosed with this in the past three months
Flu	○	○	○	○	○	○
COVID-19	○	○	○	○	○	○

How serious do you think infection with flu or COVID-19 would be (or is) to your own personal health? (If you have been diagnosed with any of these in the past three months please select that answer.)

	Not at all Serious	Not very serious	Maybe Serious	Serious	Very Serious	I have been diagnosed with this in the past three months
Flu	○	○	○	○	○	○
COVID-19	○	○	○	○	○	○

What is your understanding of where and how COVID-19 started? {freetext}

SECTION 7. Information About You.

This is the last section of the survey and is very important!

What is your age?	___________________________________
Do you live in the United States?	❍Yes<br>❍No
{If YES to live in US}<br>Please enter the first 3 numbers of your zip code.	___________________________________
{If YES to live in US}<br>We are collecting race and ethnicity information the same way the U.S. Census Bureau collects it. Which best describes your race? (You may select more than one response.)<br>Select all that apply.	❍American Indian or Alaska Native<br>❍Asian<br>❍Black or African American<br>❍Native Hawaiian or Other Pacific Islander<br>❍White<br>❍Other Race<br>❍Prefer not to answer
{If YES to live in US}<br>We are collecting race and ethnicity information the same way the U.S. Census Bureau collects it. Which best describes your ethnicity?	❍Hispanic or Latino<br>❍Not Hispanic or Latino<br>❍Prefer not to answer
{If NO to live in US}<br>In what country do you live?	
How long have you lived in the country you now reside?	❍Less than 1 year<br>❍1-3 years<br>❍4-6 years<br>❍More than 6 years
How do you identify your gender?	❍Male<br>❍Female<br>❍Non-binary<br>❍Prefer not to answer
Have you received a flu vaccine since September 1, 2019?	❍ Yes<br>❍ No<br>❍ I would like to get the flu vaccine, but am unable to for medical reasons
**{If Yes to received flu vaccine}**<br>In what month did you receive the flu vaccine?	Single month from Sept 2019 through July 2020
**{If Yes to received flu vaccine}**<br>Were you diagnosed with flu since September 1, 2019 (even though you received the vaccine)?	❍ Yes<br>❍ No<br>❍ Unsure
**{If No to received flu vaccine}**	❍ Yes

Were you diagnosed with flu since September 1, 2019	○ No<br>○ Unsure
**{Yes or No to received flu vaccine}**<br>Have you had flu-like symptoms (5 or more days of fever with any of the following: fever, cough, fatigue) since receiving your flu vaccine?	○ Yes<br>○ No
**{If Yes}** In what month(s) did you experience 5 or more days of fever with any of the following: fever, cough, or fatigue?	Select all that apply months Sept – 2019 through July 2020

This ladder represents where people stand in society. At the top of the ladder are the people who are best off, those who have the most money, most education, and best jobs. At the bottom are the people who are the worst off, those who have the least money, least education, worst jobs, or no job.	

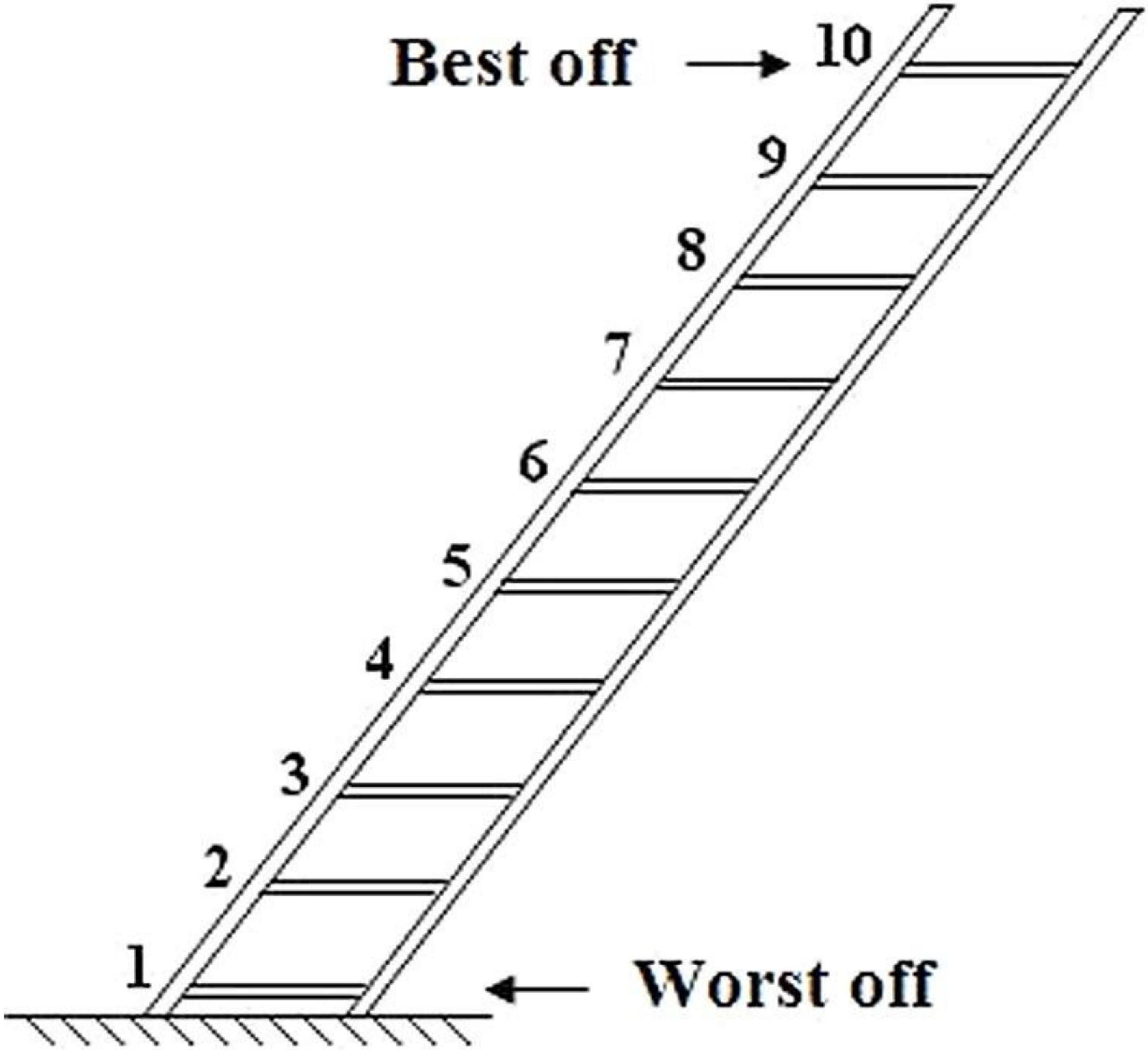

Please select the number (above) associated with the rung that best represents where you think you stand on the ladder, with 1 being the worst off and 10 being the best off.	○ 10 (Best Off)<br>○ 9<br>○ 8<br>○ 7<br>○ 6<br>○ 5<br>○ 4<br>○ 3<br>○ 2<br>○ 1 (Worst Off)

What is the highest level of education you have completed?	○Did not finish high school<br>○High School/Secondary School<br>○Some college/University

	○Associate's degree/2-year degree<br>○Bachelor's degree/4-year degree<br>○Graduate degree
Do you work in the medical profession?	○Yes<br>○No
{If Yes to Medical}<br>What best describes your PRIMARY role?<br>(select one answer)	○Physical (MD/DO, or equivalent)<br>○Certified Registered Nurse Practitioner (CRNP)<br>○Physicians Assistant (PA)<br>○Registered Nurse (RN)<br>○Administration<br>○Other clinical staff (direct patient care)<br>○Other non-clinical staff (no direct patient care)<br>○Other
{If Yes to Medical}<br>Please describe your primary role.	
{If Yes to Medical}<br>What setting best describes your clinical care setting?<br>Select all that apply.	☐Emergency department<br>☐Intensive Care Unit (ICU)<br>☐Inpatient (non-ICU)<br>☐Primary care clinic<br>☐Skilled nursing facility<br>☐Specialty care clinic<br>☐Urgent care<br>☐Not applicable<br>☐Other
{If Yes to Medical}<br>Please describe your clinical care setting.	

Have you personally been diagnosed with any of the following conditions?

	Yes	No	Prefer not to answer
Heart disease	○	○	○
Diabetes	○	○	○
Lung disease	○	○	○
Tuberculosis	○	○	○
HIV/AIDS	○	○	○
Cancer	○	○	○
Any condition that impairs your immune system (in other words, are you immunosuppressed or immunocompromised?)	○	○	○

Do you have any other comments that you wish to share about the COVID-19 pandemic?	

THANK YOU for your participation in our survey!

If you would like to learn more about WHO recommendations for COVID-19 visit:
https://www.who.int/emergencies/diseases/novel-coronavirus-2019

If you would like to learn more about Penn State University - College of Medicine research visit:
https://www.psu.edu/research
https://med.psu.edu/research

If you would like to learn more about CHIME (College of Healthcare Information Management Executives) visit:
https://chimecentral.org/

If you would like to learn more about the African American Research Collaborative visit:
https://www.africanamericanresearch.us/

If you would like to learn more about Medical College of Wisconsin research visit:
https://www.mcw.edu/research

If you would like to learn more about Mathematica visit:
https://www.mathematica.org/
